# Using Pattern Classification to Measure Adaptation to the Orientation of High Order Aberrations

**DOI:** 10.1371/journal.pone.0070856

**Published:** 2013-08-14

**Authors:** Lucie Sawides, Carlos Dorronsoro, Andrew M. Haun, Eli Peli, Susana Marcos

**Affiliations:** 1 Instituto Óptica, Consejo Superior de Investigaciones Científicas (CSIC), Madrid, Spain; 2 Schepens Eye Research Institute, Massachusetts Eye and Ear Infirmary, Harvard Medical School, Boston, Massachusetts, United States of America; University College London, United Kingdom

## Abstract

**Background:**

The image formed by the eye's optics is blurred by the ocular aberrations, specific to each eye. Recent studies demonstrated that the eye is adapted to the level of blur produced by the high order aberrations (HOA). We examined whether visual coding is also adapted to the orientation of the natural HOA of the eye.

**Methods and Findings:**

Judgments of perceived blur were measured in 5 subjects in a psychophysical procedure inspired by the “Classification Images” technique. Subjects were presented 500 pairs of images, artificially blurred with HOA from 100 real eyes (i.e. different orientations), with total blur level adjusted to match the subject's natural blur. Subjects selected the image that appeared best focused in each random pair, in a 6-choice ranked response. Images were presented through Adaptive Optics correction of the subject's aberrations. The images selected as best focused were identified as positive, the other as negative responses. The highest classified positive responses correlated more with the subject's Point Spread Function, PSF, (r = 0.47 on average) than the negative (r = 0.34) and the difference was significant for all subjects (p<0.02). Using the orientation of the best fitting ellipse of angularly averaged integrated PSF intensities (weighted by the subject's responses) we found that in 4 subjects the positive PSF response was close to the subject's natural PSF orientation (within 21 degrees on average) whereas the negative PSF response was almost perpendicularly oriented to the natural PSF (at 76 degrees on average).

**Conclusions:**

The Classification-Images inspired method is very powerful in identifying the internally coded blur of subjects. The consistent bias of the Positive PSFs towards the natural PSF in most subjects indicates that the internal code of blur appears rather specific to each subject's high order aberrations and reveals that the calibration mechanisms for normalizing blur also operate using orientation cues.

## Introduction

The perception of blur can be altered by exposure to blur introduced optically [1] or by filtering images [2], even following brief exposures to altered blur. There is solid evidence that after adapting to a sharpened image, a focused image appears blurry, and conversely, that after adapting to a blurred image a focused image appears sharpened [2], [3]. These short-term after effects reflect the visual system's adaptability, within individual observers, to changes in the spatial properties of natural images. The image formed by the eye's optics is inherently blurred by aberrations (low- and high order aberrations (HOA)) specific to each individual's eyes. Low order aberrations are normally corrected with spectacles or contact lenses, while customized refractive corrections aim at compensating also for HOA. On the other hand, certain refractive surgery treatments induce significant amounts of HOA [4], [5], while optical aids such as progressive spectacles produce significant amounts of astigmatism and field distortion [6]. A clinically relevant question is whether the visual system adapts to both correction and induction of ocular aberrations. Recent studies reveal that the eye recalibrates quickly to new patterns of astigmatism or HOA (induced or corrected) [7], [8], although whether these forms of adaptation have long-term impact on visual performance is still under investigation [8]–[11]. Meridional differences (oblique effect) [12], arising from a neural origin, have been observed on visual acuity and contrast sensitivity [13]–[16], and they likely play role on neural adaptation. So far, it appears that the internal code for blur is controlled by the overall magnitude of the subject's own aberration. The extent to which the internal code for blur may be also biased by the orientation of the aberrations pattern is debateable.

Several studies have attempted to determine what the world might look like if we could see through the eyes of another. Adaptive Optics (AO) is well-suited to addressing this question, as it allows for the manipulation of the ocular optics to produce identical retinal images in different observers. Using AO we have gained evidence that observers appear to be adapted to the blur produced by their own aberrations, as images blurred with similar magnitude of blur as the subject's own appear as best focused (unlike images blurred by lower or higher amounts of blur, which appear oversharpened or blurred, respectively [17]. The preference for the individual overall amount of blurred appears strong. However, there is some evidence that the subject may also be adapted to specific features of blur, i.e. orientation. In a recent study [18], we showed that what a subject perceived as normal is biased towards images blurred by their natural PSF as opposed those with a 90° rotation of their PSF, in agreement with a previous study showing that images blurred by the subject's Point Spread Function (PSF) were perceived as having better quality than those blurred with the same PSF but at different orientations [19]. Similarly, in a test where subjects were presented with pairs of images randomly blurred by their own PSF or someone else's PSF (selected from a set of 10 other subjects, and scaled to match the subject's own overall blur level) there was some bias towards the natural PSF, but it was weak (53±21% vs. 51±19%, on average) [18]. While prior experiments point to some role of the orientation of blur in the internal coding, they were not designed to identify the internally coded PSF.

In this study we employed a psychophysical experimental paradigm inspired by the Classification Image method. This method was first proposed by Ahumada et al. [20], [21] in audition to extract relevant features for tone detection, and more than 20 years later was applied to vision by the same authors in the study of vernier acuity tasks [22], [23]. As typically employed, the technique involves the addition of random noise to a stimulus image so that all the information that can be potentially used by a subject to perform a given task is randomly perturbed from trial to trial. Subjects make a judgement about each stimulus, e.g. whether or not a target is present. The added noises are then averaged for each of the stimulus-response categories and differenced according to whether the observer made a correct or incorrect decision. These differenced sums of random noise samples yield a profile, called the Classification Image, which is assumed to describe how the observer weighted each pixel in the stimulus to reach their trial-by-trial decisions. This technique has been used extensively to study visual strategies in a variety of visual tasks [24]: visual detection and discrimination [25]–[27], pattern recognition [28], visual filtering [29], perceived contrast of natural images [30] and adaptation to different correlated noise textures [31]. In the current study, we used a variant of the Classification Image paradigm where the variations (noise) in the image were produced by convolution with different oriented PSF while the analysis was based on the Classification Image method (for weighting and averaging) in order to extract the oriented PSF template that best matched the subject's own PSF.

## Materials and Methods

### Ethics Statement

All participants provided written informed consent. All protocols met the tenets of the Declaration of Helsinki and had been approved by the Consejo Superior de Investigaciones Científicas (CSIC) Ethical Committee. The individual photographed, for the test images of the experiment, has given written informed consent, as outlined in the PLOS consent form, to publish his photograph.

### Subjects

**Figure 1:**
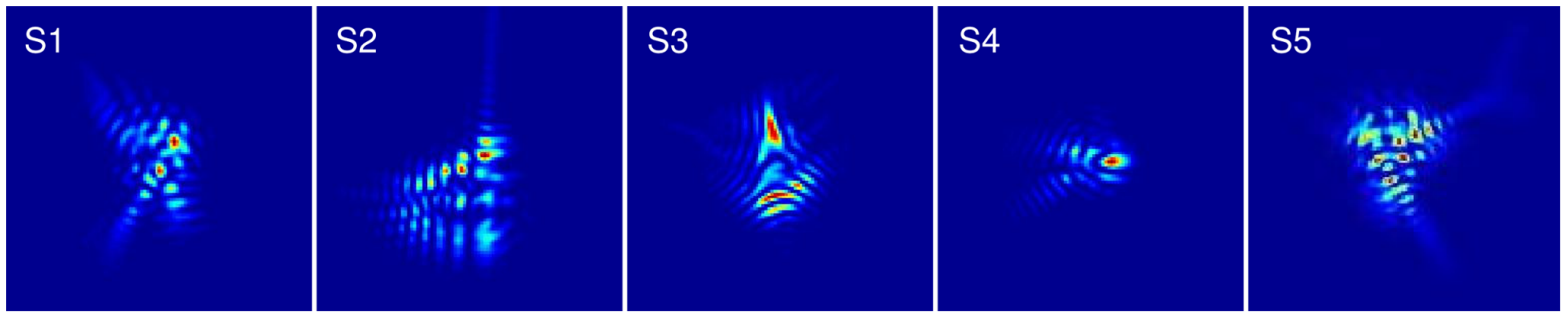
Normalized Point Spread Function (PSF) of the 5 subjects.

Five observers with prior experience in visual psychophysical tasks participated in the experiments. All had normal vision according to a clinical ophthalmological evaluation and were emmetropes or corrected ametropes. Their refractive error (without correction) was −1.85±2.59 D on average. Subject S2 performed the measurements wearing her soft contact lenses. Their natural Strehl Ratio (defined as the PSF maximum relative to the diffraction-limited PSF maximum [32] varied from 0.040 to 0.1233. Figure 1 illustrates the normalized PSF of each subject.

### Apparatus and Stimuli

Adaptive Optics (AO) allows controlling the blur level of the retinal image, and it is therefore a powerful technique to directly test neural adaptation to the subjects' own aberrations pattern.

We used a custom-developed AO system to measure and fully correct the aberrations of the subject, while viewing the stimuli. By removing the natural aberrations of the eye, all observers were exposed to identical aberration patterns and therefore any difference in the visual response must be due to neural factors. We then manipulated the retinal blur by projecting degraded images with known HOAs.

The instrument has been described in detail in previous publications [33], [34]. Illumination comes from a Super Luminescent Diode (SLD) coupled to an optical fiber (Superlum, Ireland) emitting at 827 nm. The subject's high order aberrations and astigmatism were measured with a Hartmann-Shack wavefront sensor (HASO, Imagine Eyes, France, 32×32 lenslets) and corrected by an electromagnetic deformable mirror (MIRAO, Imagine Eyes, France, 52 actuators), while defocus was corrected by a Badal optometer. Stimuli were projected on a 12×16 inches calibrated CRT Monitor, controlled by the ViSaGe psychophysical platform (Cambridge Research System, UK). The system was controlled using custom routines written in Visual C++ (Microsoft, Visual Studio), which controlled the Hartman-Shack wavefront sensor, the AO- closed loop correction of aberrations, the motorized Badal optometer, and the pupil monitoring system, and in Matlab (Mathworks, Natick, MA, USA) which controlled the ViSaGe psychophysical platform.

### Generation of the optical blur

Instead of generating the aberrations patterns with the AO-Deformable Mirror (AO-DM) [19], we manipulated the retinal blurred by projecting images blurred by convolution. The use of convolved images (observed through fully corrected optics) reduces technical complexity while still providing the intended image blur on the retina in all subjects. Also to generate the different aberration patterns (in total 1000) with the AO-DM, we would have needed to measure and generate dynamically the aberrations, thus the SLD would have been switched on during all the experiment (>3 hours). The 827-nm SLD wavelength is still visible to the subject, and the spot, superimposed to the test image, appears disturbing to the subject during performance of the psychophysical experiment. Therefore, we considered the use of convolution as the optimal solution in our experiment.

An image of a face (1.98-deg angular subtend, 480×480 pixels) was blurred by convolution with the Point Spread Function (PSF) [32] generated from 100 different wave aberrations obtained from real eyes (including 4 out of the 5 subject's wave aberrations patterns) where tilts, astigmatism and defocus were set to zero (the Zernike coefficients (HOA) that describe the aberrations of the 100 eyes are included as supporting information, Database Material S1). To generate the simulated degraded images, the original normalized Zernike coefficients of the 100 wave aberrations were scaled by a factor such that the corresponding Strehl Ratio (SR) was constant across all 100 PSFs, and matched the SR from the subject under test. Multiplying all the Zernike coefficients by a factor modifies the amount of blur while preserving the relative shape of the PSF. Each subject was then presented with images with the same overall blur level (within less than 2% deviation), but different blur orientation. Five different series of 100 images were generated, corresponding to the each of the 5 subjects participating in the experiment.

### Procedure

The high order aberrations, astigmatism and defocus of the subject were corrected, so the images were viewed under fully corrected optics. The procedures for measuring and correcting the subject's aberrations are similar to those described in detail in previous publications [33], [34]. Experiments were performed using a 5-mm artificial pupil, monocularly, and under natural viewing conditions (no cyclopegia or pupil dilation). The subject's pupil was continuously monitored to ensure proper centration.

Subjects adjusted their best subjective focus using the Badal optometer while looking at the Maltese cross on a minidisplay. The subject's astigmatism and high order aberrations were then measured and corrected in AO-closed-loop. The correction was typically achieved in 15 iterations and was deemed satisfactory when the residual wavefront error was less than 0.15 µm RMS (astigmatism and HOA). In this corrected state, the subjects were asked to again adjust the focus with the Badal system. The psychophysical measurements were performed under static correction of aberrations and the residual wavefront error was monitored (before and after each measurement) to ensure appropriate maintenance of AO-correction. A new closed-loop correction was applied if necessary (i.e. generally due to changes in eye position). On average, the RMS error (astigmatism and HOA) decreased from 0.500±0.277 µm to 0.105±0.021 µm, with an average RMS error correction of 76±8% (for 5-mm pupil diameter). The RMS for HOA-only decreased from 0.254±0.088 µm to 0.096±0.026 µm (the average percentage of HOA correction is 60±12%, across all subjects).

**Figure 2:**
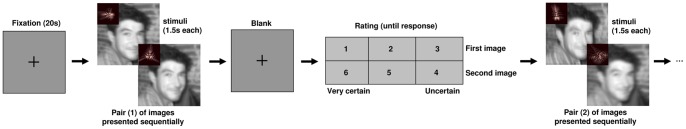
Illustration of the psychophysical experimental sequence. Presentation of two pairs of blurred images out of a total of 500 pairs followed with a response on the 6 buttons box.

In a first session, the aberrations of the participating subjects were measured to estimate their natural Strehl Ratio (needed to generate the set of images). The second session involved the Classification Images-based experiment. Subjects were presented sequentially with random pairs of images with similar overall blur level (identical PSF SR but different HOA patterns) and asked to judge which of the two images appeared better focused. The subjects used a 6-button box to respond and ranked their response with 6 choices according to their level of confidence in their judgment (from 1 meaning a high certainty that the first image presented was best-focused, and 6 meaning a high certainty that the second image of the pair was best-focused, 2 or 5 meaning moderate certainty, and 3 or 4 meaning low certainty for the first or second image, respectively). Figure 2 presents the sequence of the psychophysical experiment. Typically, the number of trials used in the Classification Image technique varies from hundreds to tens of thousands depending on the nature of the experiment. To balance this parameter with the experiment's duration, we used a total of 1000 blurred images (500 pairs, all random) presented in blocks of 50 pairs to the subject. In each pair, the images were blurred by two different HOA patterns randomly selected from among the 100 different patterns. The sequence of the psychophysical test consisted of: (1) 20 seconds adaptation to a gray field; (2) Sequential presentation of 2 blurred images (1.5 s each); (3) Re-adaptation to gray field (blank), during which the subject responded. This sequence was repeated 10 times, with 50 pairs of images presented in each run, and breaks in between runs. Images blurred with the same HOA pattern were therefore presented 10 times during the experiment. The experimental session lasted typically around 3 hours in total.

### Data analysis

The image in each pair that was judged as better focused was identified with a positive response, and the other with a negative response. The subject's PSF was compared with each PSF that resulted in a positive or negative response individually. Alternatively, the subject's PSF was compared with the average of PSFs resulting in positive or negative responses respectively. These analyses allowed extracting the features (orientation in particular) of the PSF set that best matched the subject's internal best optical blur code. The computations were carried considering either all responses, or only the 10 highest scored Positive and Negative PSFs

For the assumed optical quality metric (Strehl Ratio), all images have identical optical degradation, and therefore a random response would be consistent with natural spatial adaptation unbiased by specific features of the natural PSF of the subject e.g., the orientation. On the other hand, a consistent bias of the Positive PSFs towards the natural PSF would indicate that the internally coded blur is driven by the specific features of the subject's natural aberrations. Furthermore, if adaptation is specific to the individual aberration pattern (and not only to the overall level of blur) the average Positive PSF should match more closely the natural aberration of the subject than the Negative PSF. The analyses are therefore carried in terms of: (1) correlation of the Positive and Negative PSF (both individually and on average) with the natural PSF of the subject; (2) Orientation of the Positive and Negative PSF, in comparison with the orientation of the natural PSF.

#### Correlations of Positive and Negative PSFs with subject's natural PSF

For each subject, the Positive PSFs were weighted with +10 (high certainty, corresponding to scores of 1 or 6), +5 (moderate certainty, corresponding to scores 2 or 5) and +1 (uncertain, corresponding to scores 3 or 4). The Negative PSFs (i.e. the image not identified as positive from the pair) were given the same weight than that given to the Positive PSF of the pair, but with negative sign. This scale allowed giving a strong weight to responses with high certainty. These weights were then added over the 10 presentation instances for each test image, yielding a total score that could range from +100 to −100 (if consistently ranked as positive or negative with the highest certainty). In addition, the average Positive and Negative PSFs were calculated, by registering the centers of mass of each individual PSF (an alternative analysis using maximum intensity did not modify the results). These calculations were performed either over the 10 highest positively and negatively ranked (according to their total scores) PSFs or over all 100 weighted PSFs. Pointwise spatial correlations between the individual (or average) Positive/Negative PSFs, and the natural PSFs were calculated, and the coefficients of correlation were used to evaluate the similarity of the subject's PSF to those identified as positive or negative (both individually and averaged).

#### Analysis of PSF orientation: Sampled PSF Classification Maps and Orientation Plots

**Figure 3:**
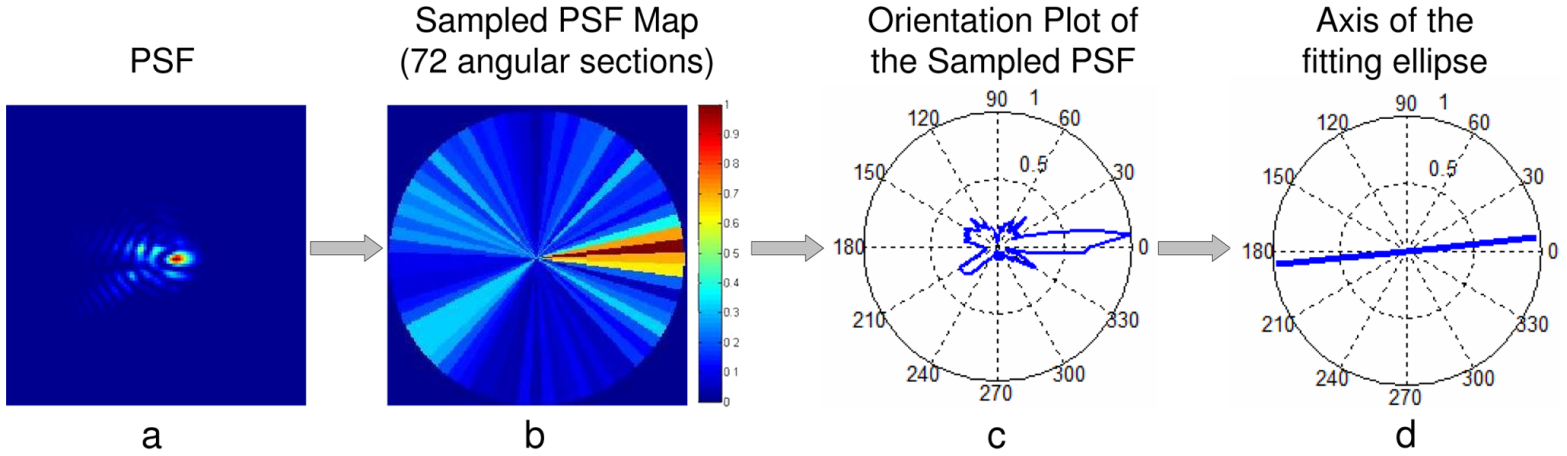
Illustration of the PSF orientation analysis. (a) Subject's PSF; (b) Sampled PSF Map in 72 angular sectors. The integrated intensity values are normalized to 1; (c) Corresponding polar plot of the Sampled PSF Map (Orientation Plot); (d) The orientation of the PSF is given by the axis of the fitting ellipse (where the angle represents the main axis of the ellipse and the line length the eccentricity e of the ellipse (e = 0.98). Data are for S4).

The subject's PSF and those identified as negative or positive were compared in terms of their orientation. To study orientations, the PSFs were sampled in 72 angular sectors (centered at the PSF center of mass, and 5-deg angles, from 0 degree centered in the first section). The analysis of PSF orientation is illustrated in Figure 3. The intensity of the PSF at a given orientation (mid-angle in each sector) was calculated as the integrated PSF intensity in each sector, and normalized to 1 (Fig. 3b), the values of the Sampled PSF plotted in polar plots (Orientation Plots, Fig. 3c). The orientation of the PSF is given by the main axis of the best fitting ellipse, with the length of the line representing the eccentricity e of the ellipse ranging from 0 (circle) to 1 (Fig. 3d).

**Figure 4:**
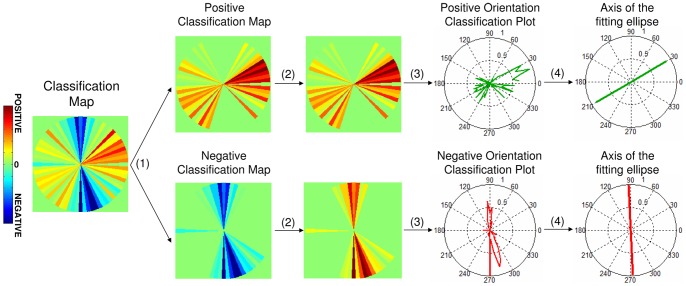
Illustration of the Classification Maps orientation analysis. (1) Construction of the Positive and Negative Classification Maps from the total Classification Map, (2) considering absolute values, (3) Polar plot representation of Positive and Negative Orientation Classification Plots, (4) main axis of the fitting ellipse and eccentricities (e = 0.88 for positive and 0.98 for negative). Example is shown for subject S4.

PSF Classification Maps are built from the subject's responses, by averaging PSFs that were given the same score (1 or 6; 2 or 5; 3 or 4) and whether considered positive or negative. In these averages, the PSFs were weighted by the factors corresponding to the certainty (high/moderate/low) of the response (+10, +5, +1; −10, −5, −1 for positive and negative responses, respectively). Classification Maps were used to extract the PSF that best matched a subject's natural PSF. describing how the subject weighted each angular section of the Sampled PSF. Also, Positive and Negative Classification Maps were computed separately, as shown in Figure 4, from the Classification Map by separating the positive and negative weights. Positive and Negative PSF Classification Maps for each subject were represented in polar plots (Orientation Classification Plots), and ellipses were fitted to these. Classification Plots represent the average orientation perceived as best or worst by the subject, respectively.

Correlations were performed between the subjects' PSF Orientation Plot and the Positive/Negative Orientation Classification Plots and used to evaluate the similarity of the subject's PSF and the Classification Maps.

The analyses rely on two assumptions: (1) The subjects' natural PSFs show a certain degree of anisotropy in orientation; (2) The 100 PSFs used in the experiment sample all orientations. The Orientation Plots of all PSFs of the sample revealed that 94% of the fitting ellipse had a well defined orientation (eccentricity>0.6), 58% had a strong orientation (eccentricity>0.9) ranging from 4 degrees to 179 degrees and 24% of the ellipses had an orientation of the long axis between 85 and 95 degrees) which generated an averaged PSF (across all the 100 PSFs of the sample) with a slight vertical orientation (eccentricity = 0.73, oriented at 85 degrees).

Correlation coefficients were used to quantify the similarity between the subject's PSF and Positive and Negative PSFs. We evaluated the sensitivity of this metric by calculating correlation coefficients of the subject PSFs with the individual PSFs of the sample, and also from the average correlation coefficients of 50 pairs of randomly selected PSFs (repeated 5 times). The Correlation coefficients between the subjects' PSFs and the 100 individual PSFs ranged from 0.055 to 0.720, and were on average for each subject: 0.462±0.093 (S1), 0.404±0.093 (S2), 0.465±0.110 (S3), 0.391±0.103 (S4), and 0.452±0.086 (S5). The coefficients of correlation of 50 pairs of randomly selected PSFs from the sample were on average 0.442±0.100 (S1), 0.424±0.101 (S2), 0.437±0.104 (S3), 0.484±0.120 (S4) and 0.408±0.097 (S5). These levels of correlations describe the distribution of sample means and represent the level of correlation that can be found by chance in this process.

#### Comparison with simulated responses

On the assumption that subjects would choose the image blurred with a more self-correlated PSF, we simulated the ideal responses of each observer to the task. The simulated observers gave positive responses for the image of each trial pair that was blurred with a PSF with a higher correlation with that of the observer's PSF. Similarly to the real experiment, we “presented” randomly 500 pairs of images, and the simulated responses were classified (and ranked) according to the correlation between the subject's PSF and the stimulus PSFs. A high certainty (1 or 6) response was given when the difference between the correlation of the PSF with one or the other image of the pair higher than 0.15, a mid certainty response (2 or 5) was given when this difference ranged between 0.15 and 0.05, and a low certainty response (3 or 4) when the difference was lower than 0.05. We performed the same analysis with the simulated data as with the human data, including weighted classification, correlation of Positive and Negative PSFs with the subject's PSF (on average and individually, and considering all responses or only the 10 top classified PSFs), and Classification Map and Orientation Plot analysis.

## Results

The subjects identified the perceived best focus image in each of the 100 pairs. All subjects showed a clear bias towards a subset of PSFs. Each image was presented 10 times to the subject, and the score was generally very repetitive. Subjects ranked only 25% of the images with a low certainty score (3 or 4), 36% were mid-certainty (2 or 5), and 39% were high-certainty (1 or 6).

### Correlations of the natural PSF with the Positive and Negative PSFs (no orientation)

**Figure 5:**
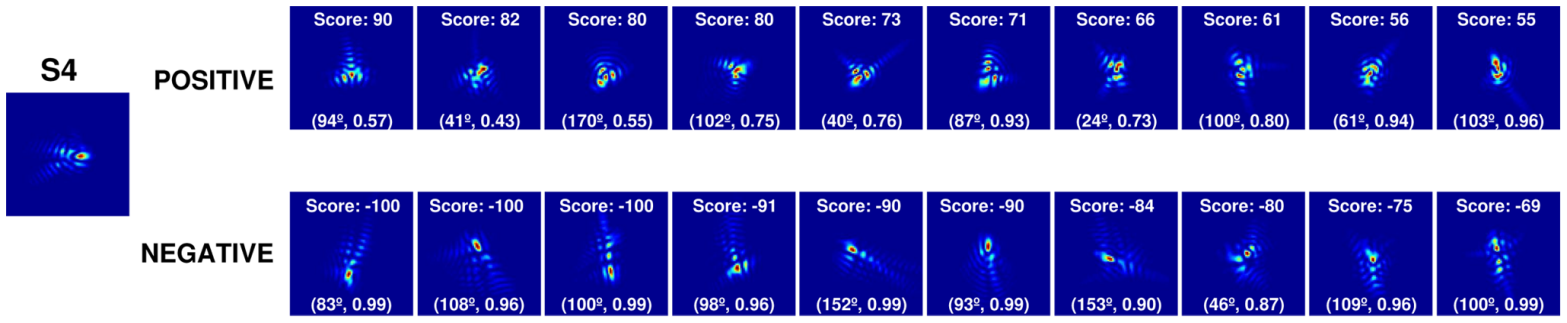
Example of the 10 top positively and negatively ranked PSFs for subject S4. The labels show the score and the parameters of the fitted ellipse (axis, eccentricity).

**Figure 6:**
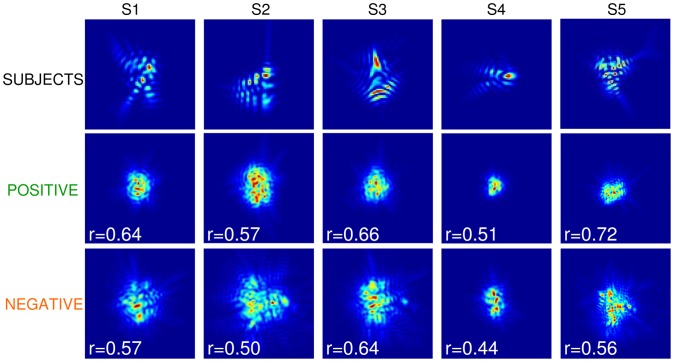
Subject's natural PSF and Positive and Negative averaged PSFs. Subject's natural PSF (first row), averaged PSFs of the 10 best positive (middle row) and of the 10 best negative (last row) for each subject. The corresponding coefficients of correlation (r) between Subject's natural PSF and the Averaged Positive and Negative PSFs are shown in each panel.

Figure 5 shows the natural PSF for one subject (S4) and 10 top Positive (scored from 90 to 55) and 10 top Negative PSFs (scored from −100 to −69). Qualitatively, the Negative PSFs tend to be vertically oriented unlike the Positive PSFs. Figure 6 shows the natural PSF of each subject, and the corresponding average Positive and Negative PSFs (average of the 10 highest scored PSFs), along with the corresponding coefficients of correlation.

**Figure 7:**
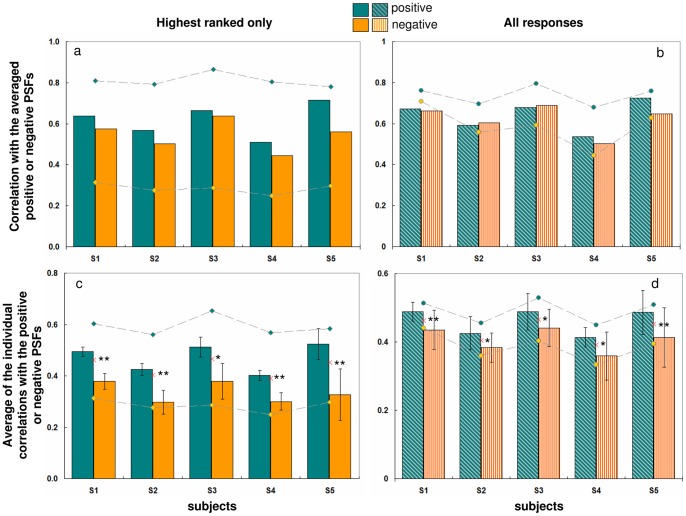
Correlations with subjects' PSF. Upper row: Correlations of the averaged Positive or Negative PSFs with the subject's PSF a) for the highest ranked only and b) for all PSFs. Lower row: Correlations of the individual Positive or Negative PSFs with the subject's PSF c) for the highest ranked PSFs only and d) for all PSFs. The red crosses show the average of all the individual correlations of the 100 PSFs with the subject's natural PSF of the eye under test. Significant differences between Positive and Negative PSFs were found in all cases; * stands for significance at p<0.05; ** p<0.005 (t-test). Dashed lines and symbols correspond to the simulated ideal responses, based on correlations with the subject's PSF. Positive responses in blue, and negative responses in yellow.

The average Positive PSFs show a higher correlation with subject's natural PSF than the average Negative PSF. The average coefficients of correlation (across subjects) were r = 0.62 for positive and r = 0.54 for negative (see inset numbers in Figure 6, and Figure 7a). Although smaller, the difference between the coefficient of correlation for Positive or Negative PSFs is still present when all Positive and Negative PSFs are averaged (instead of the 10 highest ranked only) for 3 out of 5 subjects (Figure 7b). The difference between Positive and Negative PSFs is more accentuated when the individual Positive and Negative PSFs are correlated with the subject's PSF, showing average coefficients of correlation of r = 0.47 for the Positive PSFs and r = 0.34 for the Negative (highest ranked responses, Figure 7c), and r = 0.46 and r = 0.41 for Positive and Negative (all responses, Figure 7d). These differences are statistically significant: t-test, p<0.016 for highest ranked responses; and p<0.031 for all responses.

### Analysis of PSF orientation: Sampled PSF Classification Maps and Orientation Plots

**Figure 8:**
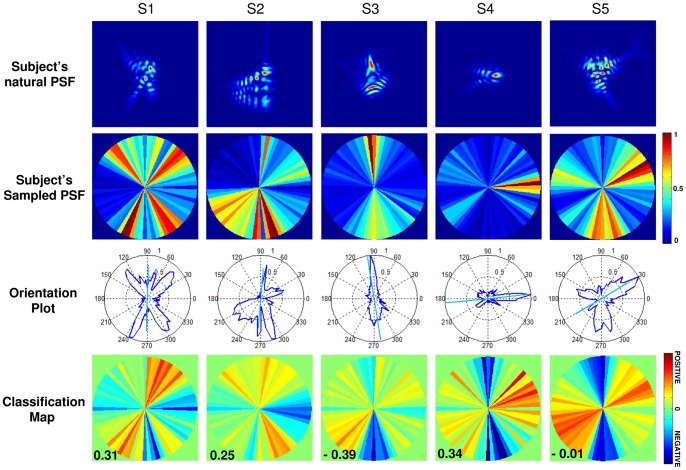
Subject's PSF and Classification Maps. (1) Subjects' natural PSF; (2) Subject's Sampled PSF in angular sectors; (3) Corresponding PSF Orientation Plot (along with the axis of the fitted ellipses and eccentricities) and (4) the Classification Maps obtained from the subject's responses and all the 100 PSFs. Correlations between the Classification Map and the subject's Sampled PSF are shown in insets.

The subjects' Sampled PSFs were correlated with the PSF Classification Maps (Figure 8). The correlation was positive in 3 subjects out of 5.

### Positive and Negative Classification Orientation Plots were compared to the subject's PSF Orientation Plots

**Figure 9:**
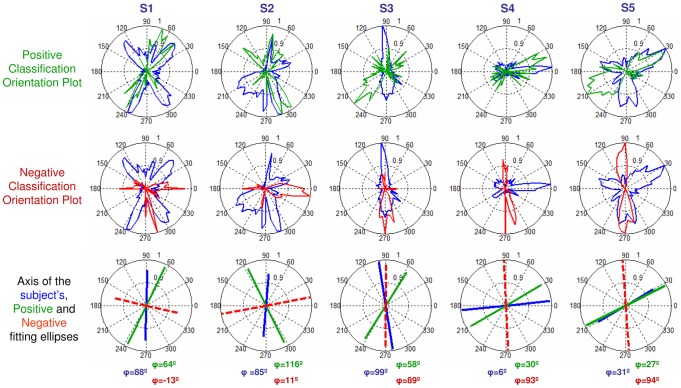
Classification Orientation Plots from subjects' responses. Positive (green) and Negative (red) Classification Orientation Plots, along with subject's natural PSF Orientation Plot (blue) for all subjects and the representation of the orientation of the fitting ellipses for the subject's PSF (blue), the positive internally coded PSF (green) and the negative internally coded PSF (dashed-red). The angle (φ) for each fitted ellipse is depicted in the corresponding graph.

(Figure 9). Except for subjects S3 and S5, there is a high degree of overlapping of the natural and Positive Classification Orientation Plots, unlike the Negative Classification Orientation Plots. In subjects S1, S2 and S4 coefficients of correlation are positive for the Positive Classification Orientation Plots (r = 0.40 (S1); r = 0.24 (S2) and r = 0.42 (S4)) and negative for the Negative Classification Orientation Plots (r = −0.35 (S1); r = −0.24 (S2) and r = −0.33 (S4)), but not for S3 (r = −0.31 and r = 0.62 for positive and negative, respectively) and S5 (r = 0.08 and r = 0.19 for positive and negative, respectively).

The bias of the PSF Classification Orientation Plots towards the natural PSF is also revealed by the orientation of the fitting ellipses (Figure 9). For all subjects except S3, the orientation of natural PSF was within 21±12 deg of the Positive Classification Orientation Plot but around 90 deg of the Negative (on average at 76±10 deg). In contrast, for S3 there was a better alignment with the Negative PSF within 10 degrees (whereas it was within 41 degrees for positive).

### Comparison with simulated responses

Theoretical simulations of the observer's responses (assuming that the responses are based on correlations between the observers' PSF and the PSF blurring the images) showed an average correlation coefficient (across subjects) of 0.81 for positive responses and 0.44 for negative responses (considering the top ten responses) and 0.74 and 0.59 respectively (considering all responses). These values set a theoretical limit to performance in the task and show a good correspondence with the average correlation values in the human subjects, as shown in Figure 7. Note how the results are similarly modulated over individual subjects in both the ideal and human performance, representing different bounds set on performance by the match between each subject's own PSF and the total stimulus set.

**Figure 10:**
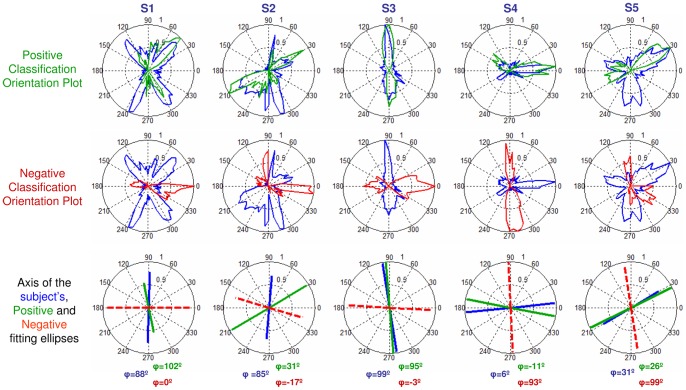
Classification Orientation Plots from simulated responses. Ideal Positive (green, upper row) and Negative (red, mid row) Classification Orientation Plots computed from the simulated responses for each subject. The axis of the fitting ellipses and their corresponding angles φ are also shown (lower row, green for positive and dashed-red for negative). The subject's natural PSF Orientation Plot (blue) and axis are shown for reference.

The simulated responses were also analysed in terms of orientation, as with the actual responses from subjects. Figure 10 compares the orientation of the Positive and Negative Classification Orientation Plots computed from the simulated responses for each subject. The orientation of the Positive PSF computed from the simulated responses closely matches, within 19°, the orientation of the subject's Orientation Plot unlike the Negative PSF oriented at 80°, on average across subjects.

Figure 10 can be compared with the orientations of the measured Positive and Negative Classification Orientation Plots in the subjects (Figure 9). There is a high similarity between theoretically simulated and actual responses. The ideal and real response orientations fall within 40° on average for the Positive PSF, and 27° for the Negative PSF. The largest discrepancy (almost 90°) occurs for S3 negative response. In subjects S4 and S5 the responses are within 12° on average.

## Discussion

A previous study showed that the internal code of blur was strongly driven by the overall blur level of the subject's HOA. The current study shows that this internal code of blur appears also to some extent to be adapted to the orientation of the natural aberrations. This confirms evidence from prior studies, which have investigated potential adaptation to the natural aberrations of the subject, using more restricted paradigms. Artal et al. [19] showed that images blurred by the natural aberrations of the subject were perceived as to have better quality than images blurred by rotating versions of the same aberration patterns. In a prior study [18], where either images blurred with the subject's aberrations or a 90° rotated version of those were used as a constant reference against images blurred by aberrations from real subjects (but similar amount of blur) showed a bias towards the subject's natural aberrations (averaging 45% versus 57% across subjects). In a companying experiment, where the aberrations of 10 subjects (including those of the subject's under test) were taken as a reference against images blurred by aberrations from real subjects (but similar amount of blur) we did not find a systematic bias towards the subject's own aberrations, with some subjects attributing higher image quality to images blurred by other subjects' aberrations (although in many cases those showed qualitatively similar orientation features than their own). The cumulative results pointed toward a weak bias toward orientation. In agreement with the previous study [18], we also found here that, although the image blurred with the subject's own PSF was often selected as best from the pair, this was not always the case (50% for S1; 70% for S2; 70% for S3; 30% for S4).

The current study used a Classification-Images inspired strategy to extract the orientation features of the PSF internally coded as producing best-perceived image quality. The fact that the internal code for blur exhibits an orientation bias indicates that not all orientations are perceived equally. In all subjects except one the orientation of the internally coded PSF matched that of the subject's own PSF (within 21 degrees, on average). In one subject, however, the orientation of the best-perceived PSF (obtained from averaging and weighting of the all subjects' responses) differed from the subject's natural aberrations (being almost perpendicular). As shown in figure 7, in this subject the correlation of the PSF averaged across all responses with the subject's natural PSF was higher for the negative than for the positive. However, the correlation between the natural PSF and the averaged PSF across the highest ranked PSFs was higher for the positive responses than for the negative. When the Positive and Negative Orientation Classification Plots were estimated only for the highest ranked responses we found that the Positive Classification Orientation Plot was in fact aligned with the subject's natural PSF (within 10 degrees) suggesting that in this subject, the perception of best-focused images was in fact very selective to her own blur orientation.

Alternatively, we tested the PSF orientation in the presence of small focus errors, and found that this particular subject experienced drastic changes in PSF orientation arising from combinations of the HOA and defocus. The internal code for blur (considering all positive responses) better aligned (within 2 degrees) with the slightly defocused (0.5 D) PSF (and within 15 degrees for 0.4D and 0.6D). In the quest for alternative reasons why the orientation for the internal code of blur and the natural's PSF of the subject disagreed in this subject (at 0 defocus/all responses), we evaluated the aberrations of the contralateral eye. To date, all previous studies addressing the extent to which subjects may be adapted to their own aberrations have investigated it from monocular measurements. However, a recent study by Kompaniez et al. [35] suggested that transfer between eyes may occur in spatial visual adaptation to blur, particularly under contingent adaptation (conflicting blur magnitude or orientation in left and right eyes). Incidentally, the subject under test showed a discrepancy in the orientation of the PSFs of both right and left eyes, although a good similarity in the amount of blur in both eyes. Measurements of the internal code for blur (Classification-Images test) in the contralateral eye revealed Classification Orientation Plots for positive and negative responses strikingly similar in both eyes (slope = 1.02, R = 0.75, p<0.05; left/right eye coefficients of correlation 0.7116 for the positive and 0.9202 for the negative maps). Despite the fact of both eyes revealing the same internal code for blur, the alignment was better with the right eye PSF than the left eye PSF, suggesting that the effect may be driven by the dominant eye in this particular subject. The extent to which the optical blur amount and orientation contribute to the internal code of blur is an extremely interesting question and remains to be elucidated.

The great similarity between the theoretically simulated responses and the actual responses in most subjects strongly supports the hypothesis that images blurred with PSFs better correlated to the subject's own are consistently perceived as to have better quality. This shows that subjects do have some sensitivity to the internal structure of their own PSFs. More specifically, the orientation of the best perceived PSF (green lines, figure 10) of an “ideal observer” (which would use correlations with its own PSF as the rule to determine best quality) closely matches the orientation of the subject's Positive PSF (green lines, figure 9).

In conclusion the Classification-Images inspired method is very powerful in identifying the internally coded blur of subjects. This pattern showed a defined orientation (generally well correlated to the subject's natural PSF), and was found to be consistent throughout time (repeated measurements in the same subject much apart) and across left and right eye of the same patient. The fact that the internal code of blur appears rather specific to each subject's high order aberrations reveals that the calibration mechanisms for normalizing blur operate using both contrast and phase/orientation cues.

## Supporting Information

Database Material S1 **Zernike coefficients (High Order Aberrations only; astigmatism and defocus are set to 0) that describe the aberrations of the 100 eyes from the VIOBIO-IO-CSIC-Patient-Database used in this study.**
(TXT)
